# Characterization and Application of Pomegranate Epicarp Extracts as Functional Ingredients in a Typical Brazilian Pastry Product

**DOI:** 10.3390/molecules25071481

**Published:** 2020-03-25

**Authors:** Felipe da Silva Veloso, Cristina Caleja, Ricardo C. Calhelha, Tânia C. S. Pires, Maria José Alves, Lillian Barros, Aziza K. Genena, João C. M. Barreira, Isabel C. F. R. Ferreira

**Affiliations:** 1 Centro de Investigação de Montanha (CIMO), Instituto Politécnico de Bragança, Campus de Santa Apolónia, 5300-253 Bragança, Portugal; felipeveloso1@live.com (F.d.S.V.); ccaleja@ipb.pt (C.C.); calhelha@ipb.pt (R.C.C.); tania.pires@ipb.pt (T.C.S.P.); maria.alves@ipb.pt (M.J.A.); lillian@ipb.pt (L.B.); 2 Departamento Acadêmico de Alimentos (DAALM), Universidade Tecnológica Federal do Paraná, Campus Medianeira, Parana 85884-000, Brazil; azizakg@utfpr.edu.br

**Keywords:** *Punica granatum* L., phenolic compounds, pectin, bioactivity, food application

## Abstract

Currently, there is a clear tendency to incorporate natural ingredients into food and pharmaceutical formulations. Besides being well-accepted by consumers, these ingredients have less adverse side effects than their artificial counterparts. The pomegranate processing industry produces large quantities of by-products that are discarded as bio-residues, despite containing bioactive compounds. Accordingly, the epicarp of two pomegranate varieties (*Mollar de Elche* and *Purple Queen*) was tested as a potential source of bioactive compounds with food application. The phenolic profile was identified by HPLC–DAD–ESI/MS, revealing fourteen phenolic compounds in both varieties (*Purple Queen* showed also three anthocyanins), with punicalagin isomers as the major compounds. Nonetheless, *Mollar de Elche* presented greater antioxidant and antibacterial activities. Despite this result, *Purple Queen* was selected to be tested as a new natural colouring and functionalizing ingredient in a Brazilian pastry product. The incorporation of the selected extract maintained the nutritional profile and provided a higher antioxidant activity compared to the traditional product. In this way, this work confirmed the possible use of pomegranate epicarp as a natural ingredient in the food industry, conferring dyeing and functionalizing effects, and anticipating a possible valorisation of this bio-residue.

## 1. Introduction

The utilization of products remaining from industrial processing of vegetal products represents an approach with high probability of success, besides holding ecological advantages, considering that the intense growth of the global horticultural sector has produced alarming amounts of discards [1]. However, these bio-residues often contain high-value compounds such as polyphenols, vitamins and fibres [2].

*Punica granatum* L. (pomegranate), is currently placed in the family Lythraceae, although it was previously included in the Punicaceae [3]. It is ranked among the twenty fruits with highest production levels, and its phenolic compounds have been extensively studied [4]. More than 150 phenolic compounds have been identified in pomegranates, including ellagitannins, anthocyanins, flavonols (e.g., quercetin), phenolic acid (e.g., caffeic acid, chlorogenic acid, gallic acid and quinic acid) and ellagic acid [5,6]. Actually, punicalin and punicalagin were named after the pomegranate genus, due to the high concentrations of these compounds in this fruit [7,8]. Some of the phenolic compounds present in the pomegranate composition are described as being responsible for its significant biological activity, namely anti-inflammatory action, prevention against certain types of cancer [9], hypoglycaemic and hypocholesterolaemic action [10,11], or preventive action against neurodegenerative diseases [12].

Considering that more than 50% of the whole fruit is discarded as bio-residue, pomegranate has high potential as a source of bioactive compounds for different uses [13]. In addition, the pomegranate epicarp has a concentration of phenolic compounds (essentially tannins, catechins, gallocatechins and prodelfinidines) as much as three times higher than the pulp [14]. Likewise, the percentage of anthocyanins found in the epicarp is very relevant, namely, cyanidin-3,5-*O*- diglucoside, pelargonidin-3,5-*O*-diglucoside, pelargonidin-3-*O*-glucoside, cyanidin-3-*O*- glucoside and cyanidin-3-*O*-rutinoside [5]. In this sense, the food industry has been exploring this type of matrix as a source of natural ingredients with possible application in new products. Accordingly, pomegranate was tested as a new natural colourant and bioactive ingredient, which may represent an effective way to valorise this this bio-residue.

## 2. Results and Discussion

### 2.1. Phenolic Characterization of Extracts of Pomegranate Epicarp

From the point of view of bioactive compounds content, pomegranate is particularly interesting for its phenolic compound profile, already described as exerting excellent physiological activities (antioxidant, antimicrobial and anti-inflammatory) and disease-preventive actions [9,15]. Phenolic compounds are essentially present in the epicarp, a non-edible component of the fruit which may represent more than 50% of its total weight [13]. In this study, two varieties (sold as *Mollar de Elche* and *Purple Queen*) were selected for their different phenotypic characteristics and commercial diffusion. Besides the potential differences induced by each variety, the influence of maceration solvent (water, ethanol or an equal w/w mixture of the former) used in the maceration was also verified.

The phenolic profile of *Mollar de Elche* included fourteen compounds, while that of *Purple Queen* presented seventeen (including three anthocyanins). Table 1 describes the chromatographic parameters and spectral data that were considered in the identification of the compounds present in extracts of pomegranate epicarps. The phenolic profile and quantification of the ethanolic, aqueous and hydroalcoholic extracts of both varieties are presented in Table 2. The compounds were identified considering the chromatographic and spectral data previously described in the literature for pomegranate [5,6,8,15,16,17,18,19,20,21]. The identified compounds included five phenolic acids, seven hydrolysable tannins and five flavonoids (two flavonols and three glycosylated anthocyanins).

Among phenolic acids (or derivatives), peak 1 was identified as galloylglucose, having a molecular ion [M − H]^−^ at *m*/*z* 331, which produced an MS^2^ fragment at *m*/*z* 169 ([M − 162]^−^ corresponding to the loss of one hexose relative to gallic acid. Peak 12 was identified as ellagic acid according to the mass and UV spectra and retention times of a commercial standard. On the other hand, peaks 7 ([M − H]^−^, *m*/*z* 463), 10 ([M − H]^−^, *m*/*z* 433) and 11 ([M − H]^−^, *m*/*z* 447) presented UV–Vis and mass spectra characteristic of ellagic acid derivatives, producing a major MS^2^ fragment at *m*/*z* 301 (ellagic acid) after the release of hexosyl units (−162 mu, peak 7), pentosyl (−132 mu, peak 10), or rhamnosyl (−146 mu, peak 11). These phenolic acid derivatives were previously described in pomegranate extracts [8,16,17].

The compounds identified as ellagitannins corresponded to peaks 2–6, 8 and 9. Peaks 3 and 4, in particular, were identified as isomers I and II of punicalagin, based on the detected pseudomolecular ion ([M − H]^−^ at *m*/*z* 1083) and the fragmentation standard previously described by Ambigaipalan et al. [16], Qu et al. [20] and Mena et al. [6]. These molecules were also indicated as being the majority ellagitannins described in pomegranate juice, which was also observed in the present study (see next Section). Peak 2 ([M − H]^−^ at *m*/*z* 783) presented fragments at *m*/*z* 481 and 301, allowing its identification as pedunculagin (bis-HHDP-glucose) [6]. The mass spectral characteristics of peak 3 ([M − H]^−^ a *m*/*z* 633, fragments *m*/*z* 463 and 301) coincide with galloyl-HHDP-glucose, also described in pomegranate [5,6]. Similarly, peak 6 was identified as digalloyl-HHDP-glucose, another common pomegranate compound [6,16,17,21]. Peaks 8 and 9 presented the same molecular ion ([M − H]^−^ at *m*/*z* 633), which in this case was attributed to granatin B (digalloyl-HHDP-DHHDP-hexose) and identified as two distinct derivatives of this compound [6,17,21].

In what concerns flavonoids, peaks 13 and 14 were identified as kaempferol-3-*O*-rutinoside and kaempferol-3-*O*-glucoside, respectively, considering their maximum absorbance (λ_max_) at 348 nm, the characteristic fragmentation standard of glycosylated kaempferol derivatives, UV spectrum and retention time; both compounds were previously described in pomegranate [6,18].

According to the quantification results, the major compounds in *Mollar de Elche* were the two isomers of punicalagin and galloyl-HHDP-glucose, and this tendency was verified in the three extracts. Generally, the extracts obtained with ethanol presented significantly higher contents. The higher efficacy of ethanol for extracting phenolic compounds from pomegranate epicarp was also verified in the case of *Purple Queen*. Likewise, the major compounds in *Purple Queen* variety were the two isomers of punicalagin, although in lower quantities than those obtained in *Mollar de Elche*.

On the other hand, anthocyanins, specifically cyanidin-3,5-*O*-diglucoside, cyanidin-3-*O*- glucoside and pelargonidin-3-*O*-glucoside, were only detected in *Purple Queen*. In the case of this subgroup, the aqueous extract allowed the greatest yield, which is in agreement with the greater polarity of this type of compounds. Cyanidin-3-*O*-glucoside and pelargonidin-3-*O*-glucoside were also identified after comparison of the chromatographic and spectral data with commercial standards. A third anthocyanin ([M-H]^−^ at *m*/*z* 611) was identified as cyanidin-3,5-*O*-diglucoside, based on its fragmentation standard. The three anthocyanins identified are also characteristic of pomegranate [5,6,8,17].

### 2.2. Characterization of Bioactivity of Extracts of Pomegranate Epicarp

As could somehow be anticipated, considering the contents of phenolic compounds described in the previous section, all the extracts presented a significant antioxidant activity. In the case of *Mollar de Elche* pomegranate, the highest activity was measured in the aqueous extract (10 μg/mL), while in *Purple Queen* the extract with the greatest lipid peroxidation inhibition capacity was the hydroalcoholic (10 μg/mL). Thus, there was no direct correlation with the levels of phenolic compounds which, as described in the previous section, were higher in the ethanolic extract, indicating that other compounds with antioxidant activity may be present in the extracts. Even so, the lowest EC_50_ values were obtained in *Mollar de Elche*, which correlates with the highest concentrations of phenolic compounds quantified in this variety.

In addition to antioxidant activity, the cytotoxicity evaluated in *Mollar de Elche* epicarp extracts was also superior to that observed in *Purple Queen* extracts. However, in both cases, a greater effectiveness of the ethanolic extracts was verified. The cell line with the highest growth inhibition (Table 3) was HeLa (91 μg/mL for *Mollar de Elche* ethanolic extract, 153 μg/mL for *Purple Queen* ethanolic extract), whereas the one that showed to be less affected was NCI-H460 (194 μg/mL for *Mollar de Elche* ethanolic extract, 268 μg/mL for *Purple Queen* ethanolic extract). This differentiation in inhibitory capacity was verified independently of the extract type. In the case of hepatotoxicity, GI_50_ values were higher in all cases, which can be considered as a good result, despite the lower proliferation rate that characterizes this cell line, when compared with the evaluated tumour cell lines.

According to Table 4, all samples showed inhibitory activity against the analysed microorganisms. However, they did not show bactericidal capacity up to the maximum tested concentration of 20 mg/mL.

In general, Gram-positive bacteria were the most sensitive, as lower concentrations (MIC = 0.625–5 mg/mL) of extracts were required to inhibit their growth. Within these bacteria, MRSA showed higher sensitivity (MIC values of 0.625–1.25 mg/mL), followed by *E. faecalis* (MIC = 0.625–2.5 mg/mL) and *L. monocytogenes* (MIC = 2.5–5 mg/mL); the measured activity was higher than that reported by Alexandre et al. (MRSA: MIC = 3.91 mg/mL; *L. monocytogenes*: MIC = 15.63 mg/mL) [22].

For Gram-negative bacteria, *M. morganii*, *P. mirabilis* and *E. coli* were the most sensitive (MIC = 1.25–2.5 mg/mL), followed by *K. pneumoniae* (MIC = 1.25–5 mg/mL), and *P. aeruginosa*, which proved to be the most resistant (MIC in the range of 10–20 mg/mL). Fawole et al. [4] reported lower MIC values with methanolic extracts (*E. coli*: 0.78 mg/mL; *K. pneumoniae*: 0.39 mg/mL), but higher MIC values for aqueous extracts (12.50 mg/mL for both bacteria).

Comparing the pomegranate varieties, *Mollar de Elche* presented higher antibacterial activity (for all extracts), as evidenced by lower MIC values. The best extract analysed was obtained with ethanol (MIC of 0.625 and 1.25 mg/mL in most cases), which agrees with the higher concentration of phenolic compounds in *Mollar de Elche*. In contrast, the aqueous extract from *Purple Queen* epicarp showed the weakest antibacterial activity (MIC = 1.25–20 mg/mL).

It is important to note that the tested samples showed lower MIC than ampicillin against some bacteria. In the study of the inhibitory effect against *K. pneumoniae*, for example, 10 mg/mL of ampicillin was required, while the extracts tested showed the same capacity at lower concentrations (1.25 to 5 mg/mL). Similarly, a MIC of 20 mg/mL was required for *M. morganii*, while the extracts exerted the same effect at concentrations among 1.25 and 2.5 mg/mL. For *P. aeruginosa*, ampicillin had no inhibitory activity up to 20 mg/mL, whereas the extracts inhibited this species with concentrations of 10 to 20 mg/mL. These results interesting, given the increasing resistance of these microorganisms to the antibiotics used in clinical practice. Thus, the search for natural alternatives with similar or better effectiveness is a global priority in the fight against these resistant bacteria.

Some studies have highlighted the high number of compounds present in the pomegranate peel, which has aroused great curiosity, mainly by the food industry, aiming using a bio-waste with high application potential [23]. The presence of a high concentration of phenolic compounds has been justifying the different bioactivities revealed by the skin of different varieties of pomegranate. A study by Gullon et al. [24] revealed the antimicrobial ability of pomegranate peel (Mollar de Elche) for all of the microorganisms studied both Gram positive (*S. aureus*, *L. monocytogenes* and *L. innocua*) and Gram negative (*E. coli*, *P. aeruginosa* and *Salmonella* sp.). Similarly, the antioxidant activity of the peels has also been highlighted. In particular Derakhshan et al. [23]., highlighted greater antioxidant activity in the peels when compared to seed and juice of their extracts.

### 2.3. Characterization of New Formulations of “Casadinhos”

In general, the different extracts (mainly ethanolic extracts) obtained from the epicarp of both pomegranate varieties represent a source of phenolic compounds that allow it to exhibit excellent bioactive properties with potential application in the food industry. However, when developing novel foods, the end product may not exhibit certain types of bioactivity, e.g., antimicrobial, for the well-known reasons associated with the appearance of multi-resistant microbial strains, or cytotoxic effects, since this particularity could cause undesirable effects on normal cell proliferation in the body (for example along the gastrointestinal epithelium). For this reason, associated with the greater colouring ability of the extracts obtained from *Purple Queen* epicarp, the ethanolic extracts of this variety were used in the preparation of a traditional Brazilian pastry product, called *casadinhos*.

Using *Mollar de Elche* epicarp would not provide such an obvious change in the appearance of the products, besides increasing the possibility of obtaining a final product with undesired bioactive features (cytotoxic and antimicrobial). In order to increase the usefulness of pomegranate, a third formulation of this product was prepared in which the traditionally used filling, guava jelly, was substituted for a jelly prepared from pomegranate juice and pectin extracted from the epicarp.

In summary, three formulations were prepared: (i) traditional (CT), (ii) *casadinhos* with incorporation of pomegranate epicarp extract and guava filling (CEC), and (iii) *casadinhos* incorporating pomegranate epicarp extract and pomegranate jelly filling (CECP).

The different formulations were compared for their nutritional composition, free sugars and fatty acids profile and the colour parameters: L*, a* and b*. In order to verify the functionalization of the product, its ability to inhibit lipid peroxidation was also evaluated.

It should be noted that, in any case, two factors of statistical variability were considered, namely the formulation (F) and the storage time (ST). Thus, in addition to assessing the significance of the changes induced by each individual factor, it is also necessary to verify if the two factors have significant (*p*-value < 0.05) interaction (F × ST). Presenting the results in this way implies that the value for each storage period is obtained from the average of the values of the three formulations for that period, and also, obviously, that the result obtained for each formulation is the result of the average of the three storage times for that formulation (this justifies some relatively high standard deviations values).

#### 2.3.1. Nutritional Composition and Sugar Profile of “Casadinhos”

Table 5 shows the average values, in g/100 g of fresh product, obtained for the nutritional composition, free sugars and energy value of the analysed formulations. In general, carbohydrates were the most abundant component (≈74%), followed by fat (≈20%) and water (≈3–7%). In relation to the effects of each individual factor, while there were significant differences between the formulations for all parameters, the same was verified for ST only in the case of water, carbohydrates, sucrose, ash and energy content. The interaction was significant for all parameters, except for protein content, justifying why the classification obtained from the multiple comparison tests was performed only in the case of this parameter, and in relation to the effect of the formulation, which presented higher levels in the non-stored samples and in CECP. In relation to the other parameters, it was possible to obtain some conclusions from the estimated marginal means. For instance, fructose and glucose presented lower values in CT samples, while sucrose was detected in lower concentrations in CEPC. On the other hand, CEC had the lowest values of carbohydrates (73 g/100 g), fat (19 g/100 g) and energy (469 kcal/100 g) being, on the other hand, those that registered higher levels of water (7 g/100 g). Regarding ST effect, the induced differences were not so evident, except for the lower carbohydrate content (72 g/100 g) observed in non-stored samples. The incorporation of natural ingredients in different food matrices has been tested in order to verify its potential application as substitutes for artificial additives. Some studies on different pastry products have proven that this same application does not cause significant changes in the food matrix [25,26].

#### 2.3.2. Fatty Acids Profile of “Casadinhos”

As a previous note, it should be highlighted that only fatty acids with percentages greater than 1% for at least one of the formulations or storage times were tabulated.

Once again, there was a significant interaction between the two factors (F × ST) for almost all cases, except for the percentage of C15:0 (*p* = 0.355), which did not show differences for each single factor. However, in the case of fatty acids, ST effect was more significant than that of the formulation (Table 5), as can be verified by the number of fatty acids with significant differences for each factor.

Despite these differences, all formulations presented similar profiles, characterized by high percentages of palmitic acid (≈30%) and oleic acid (≈21%), the major fatty acids.

Similarly, and despite the observed differences, it was not possible to identify unequivocal trends resulting from the individual effects of each factor, regardless of the statistically significant differences that were detected in some cases.

Thus, it can be concluded that the addition of the extracts in the dough and using pomegranate jelly as filling did not cause significant changes in the fatty acid profile of the traditional formulation of *casadinhos*.

#### 2.3.3. Colorimetric Parameters and Textural Characteristics of “Casadinhos”

In addition to the nutritional aspects and the individual components discussed in the previous sections, the analysis of the colour parameters (Table 5), namely L*, a* and b*, has an increased relevance, considering the objective of achieving a more appealing appearance for this food product.

The changes observed in physical properties, namely in colorimetric parameters and structural characteristics, resulted, again, from the cooperative action of both factors, since their interaction was always significant.

Regarding the individual effect of each factor, a higher influence of the formulation was observed, since ST only caused significant changes in fracturability (*p* = 0.034) and chewiness (*p* = 0.004). Therefore, it was once again necessary to observe the graphs of the estimated marginal means to infer some trends.

In relation to lightness, the addition of ethanolic extract of *Purple Queen* caused a slight lowering, having, on the other hand, and as expected, increased the intensity of the red hue (a*). As for parameter b*, the CEC formulation presented statistically lower values, although the magnitude of the difference is negligible. It should be noted that the small variations in the colour hue detected may also be due to the Maillard reactions. The result of these reactions is the golden aspect of the food when toasted or caramelized. This reaction occurs between amino acids or proteins (amine groups) and sugars (carbonyl groups) when the food is made, leading to the production of melanoidins [27].

The *casadinhos* prepared with the incorporation of pomegranate extract also presented lower hardness and fracturability. Thus, it is consistent with chewiness (the energy needed to chew food), which is superior in the case of the traditional formulation, raising the possibility of increasing the range of consumers.

#### 2.3.4. Bioactivity of the “Casadinhos”

In addition to the appearance and texture of samples added with pomegranate extract, it was also important to verify if these new products maintained the antioxidant activity determined in the extracts, which would be an advantageous feature.

In the inhibition of thiobarbituric acid reactive substances, the traditional formulation showed EC_50_ = 2268 ± 714 μg/mL, while CEC and CECP presented incomparably lower values: EC_50_ = 79 ± 11 μg/mL and EC_50_ = 67 ± 21 μg/mL, respectively. In addition to this important improvement in the antioxidant activity, it was also possible to verify that, while there was a loss of the already weak activity of the formulation over the storage time, in the cases of CEC and CECP, the antioxidant activity remained unchanged over the 14 days of storage. The incorporation of antioxidant activity has been proven by other authors, who have even proved the effectiveness of natural ingredients compared to artificial additives [28,29].

## 3. Materials and Methods

### 3.1. Preparation of Samples

Two different types of pomegranates sold as *Purple Queen* (three samples of red pomegranate) and *Mollar of Elche* (three samples of yellow pomegranate) were purchased from a local market in Bragança, Portugal. The epicarp was finely separated from the pulp and both were frozen and then lyophilized and stored in a desiccator at room temperature (average 25 °C), protected from light, until further analysis.

### 3.2. Extracts Preparation

The lyophilized samples (1 g) of both varieties were submitted to maceration at room temperature with 30 mL of different solvents (water, ethanol or ethanol:water 50:50, *v/v*), during 1 h with agitation (150 rpm). Subsequently, extracts were filtered through a filter paper (Whatman No. 4) and the retained residue was re-extracted under the same conditions. Finally, the alcoholic fraction of the extracts obtained was evaporated under reduced pressure (rotary evaporator, Büchi R-210, Flawil, Switzerland) and lyophilized (FreeZone 4.5, Labconco, Kansas City, MO, USA).

#### 3.2.1. Identification and Quantification of Phenolic Compounds

The phenolic compounds (non-anthocyanin and anthocyanin compounds) were separated, identified, and quantified following a previously optimized methodology [30,31] and using a Dionex Ultimate 3000 UPLC system (Thermo Scientific, San Jose, CA, USA). The detection was performed with a DAD and a mass spectrometer (LTQ XL mass spectrometer, Thermo Finnigan, San Jose, CA, USA) working in negative mode for non-anthocyanin compounds and positive mode for anthocyanin compounds.

Analytical curves (200–5 µg/mL) of the available phenolic standards were constructed based on the UV-Vis signal: gallic acid (y = 365.2x − 38.923, R^2^ = 0.999); ellagic acid (y = 38466x + 35.44, R^2^ = 0.9994); kaempferol-3-*O*-rutinoside (y = 182.94x + 96.644, R^2^ = 0.997); kaempferol-3-*O*-glucoside (y = 236.33x + 70.006, R^2^ = 0.999); cyanidin-3-*O*-glucoside (y = 134578x – 3 × 10^6^; R² = 0.9986) and pelargonidin-3-*O*-glucoside (y = 61493x – 628875; R² = 0.9957). Results were expressed in mg/g of dry extract.

#### 3.2.2. Evaluation of Extracts’ Antioxidant Activity

The lyophilized samples were re-dissolved in ethanol and successively diluted (5–500 μg/mL) to allow determining the corresponding EC_50_ values. The lipid peroxidation inhibition in porcine (*Sus domesticus*) brain homogenates was evaluated by the decrease in thiobarbituric acid reactive substances (TBARS) following the protocol described by Barros et al. [32]. The results were expressed in EC_50_ values (sample concentration providing 50% of antioxidant activity or 0.5 of absorbance in the reducing power assay) and Trolox was used as the standard.

#### 3.2.3. Evaluation of Extracts’ Toxicity

All the extracts were tested in four human tumour cell lines: MCF- 7 (breast adenocarcinoma), NCI-H460 (non-small cell lung cancer), HeLa (cervical carcinoma) and HepG2 (hepatocellular carcinoma), using the sulforhodamine B assay to measure the cell growth inhibition [33]. The hepatotoxicity was measured by using a freshly harvested porcine liver cell culture, PLP2 (acquired from certified slaughterhouses) [32].

In both assays, a phase contrast microscope was used to monitor the growth of cell cultures, which were sub-cultured and plated in 96-well plates (density of 1.0x10^4^ cells/well). Dulbecco’s modified Eagle’s medium (DMEM) supplemented with FBS (10%), penicillin (100 U/mL) and streptomycin (100 μg/mL) were used. As a positive control, ellipticin was used. The results were expressed as GI_50_ values (μg/mL).

#### 3.2.4. Evaluation of Extracts’ Antibacterial Activity

The bacterial strains were clinical isolates obtained from patients hospitalized in various departments at the Hospital Center of Trás-os-Montes and Alto Douro (Vila Real, Portugal). Five Gram-negative bacteria (*Escherichia coli*, *Proteus mirabilis*, *Klebsiella pneumoniae*, *Pseudomonas aeruginosa* and *Morganella morganii*) and three Gram-positive bacteria (*Enterococcus faecalis*, *Listeria monocytogenes* and methicillin-resistant *Staphylococcus aureus* (MRSA)) were tested. All these microorganisms were incubated at 37 °C in appropriate fresh medium for 24 h before analysis to maintain the exponential growth phase.

The MIC determinations on all bacteria were conducted using colorimetric assay according to described by Pires et al. [34]. Ampicillin and Imipenem were used for all Gram-negative bacteria tested and *Listeria monocytogenes*. Ampicillin and vancomycin were selected for *E. faecalis* and MRSA. For the determination of MBC, 10 μL of liquid from each well that showed no change in colour was plated on solid medium, Blood agar (7% sheep blood) and incubated at 37 °C for 24 h.

### 3.3. Incorporation of Natural Ingredient in a Traditional Brazilian Bakery Product

*Purple Queen* was selected to be tested as a new natural colouring and functionalizing ingredient and three groups of samples were prepared: (i) control samples (*casadinhos* with traditional cake dough and guava jam); (ii) samples with functional dough (*casadinhos* with cake dough functionalized with red pomegranate epicarp ethanolic extract and guava jam); (iii) samples with functional dough and jam (*casadinhos* with cake dough functionalized with red pomegranate epicarp ethanolic extract and pomegranate jelly).

*Casadinhos* dough was prepared by mixing wheat flour, sugar and butter. After that in both functionalized groups the red pomegranate epicarp extract was added to the mixture. The dough was then divided into small spherical portions and baked at 180 °C for 15 min. The filling was placed directly between two cooked portions. Guava jam was purchased at a local store. The pomegranate jam was prepared with water, lemon, sugar and juice of a red pomegranate. The mixture was warmed and stirred slightly for about two hours.

All samples were lyophilized, finely crushed and analysed, in triplicate, immediately after preparation and after seven and fourteen days of storage (at room temperature and packed in a sealed plastic bags covered with aluminium paper).

#### 3.3.1. Evaluation of Colour Parameters and Texture of “Casadinhos” during Storage Time

The colour of the samples was measured using a colorimeter (model CR-400, Konica Minolta Sensing Inc., Tokyo, Japan). The illuminate C was used and a diaphragm aperture of 8 mm and previously calibrated against a standard white tile. The CIE L* (lightness), a* (greenness/redness), b* (blueness/yellowness) colour space values were registered using a data software ‘‘Spectra Magic Nx” (version CM-S100W 2.03.0006) [35]. The texture was determined using a TA-XT plus Texture Analyser implemented with the Exponent software version 6.1.11.0 (Stable Micro System, London, UK) with an acrylic disk (40 mm) in order to measure different parameters: hardness, adhesiveness, resilience, cohesion, elasticity, gum and chewability.

#### 3.3.2. Nutritional Composition

The contents of protein, fat, carbohydrates and ash, were determined following the AOAC methods [36] and following a procedure previously reported by Barros et al. [32]. Total energy was calculated following the equation:

Energy (kcal) = 4 × (g protein + g carbohydrates) + 9 × (g fat)(1)

#### 3.3.3. Chemical Composition

##### Free Sugars Present in Epicarps of Both Varieties

One gram of dried sample was mixed with melezitose (internal standard (IS) 5 mg/mL), and extracted with 40 mL of ethanol:water (80:20) 80 °C for 30 min. After that, the suspension was centrifuged (Centurion K24OR refrigerated centrifuge, West Sussex, UK) at 15,000× *g* for 10 min and the supernatant was concentrated at 60 °C under reduced pressure and defatted three times with 10 mL of ethyl ether, successively. The solid residues were dissolved in water to a final volume of 5 mL and filtered through 0.2 μm Whatman nylon filters. HPLC coupled to a refraction index (RI) detector was used to determine the free sugars following a previously described procedure [35]. The free sugars were identified by comparison with standards and further quantified considering the internal standard.

##### Fatty Acids in Pulp and Epicarp of Both Varieties

The fatty acids were determined by gas chromatography coupled with a flame ionization detector (GC–FID, DANI model GC 1000, Contone, Switzerland), following a procedure previously described [32]. The results were expressed as relative percentage of each fatty acid.

#### 3.3.4. Evaluation of Antioxidant Activity

The lyophilized samples (3 g) were extracted with ethanol/water (80:20) at room temperature during 1 h under stirring. The extract was filtered with Whatman paper filter Nº 4 (Sigma-Aldrich, St Louis, MO, USA) and the remaining solid residue subjected to an additional extraction at the same conditions. The extracts were evaporated under reduced pressure in a rotatory evaporator until complete removal of methanol. Finally, the evaporated extract was dissolved in methanol at a concentration of 50 mg/mL and submitted to TBARS previously described in Section 3.2.2.

### 3.4. Statistical Analysis

For each extract (aqueous, ethanolic or hydroalcoholic), three independent samples were analysed in triplicate. Likewise, from each *casadinho* formulation, three independent samples were also selected, and each sample was analysed in triplicate. Data were expressed as mean±standard deviation. The statistical tests were applied considering a value of α = 0.05 (95% confidence) using the IBM SPSS Statistics for Windows software version 25.0 (IBM Corp., New York, NY, USA).

An analysis of variance (ANOVA) was performed, based on the Tukey test (when homoscedasticity of the distributions was verified) or the Tamhane’s T2 test (heteroscedastic distributions) to classify the statistical differences between the different parameters evaluated in each of the extracts. Compliance with the ANOVA requirements, specifically the normality of the distribution of results and the homogeneity of variances, was verified through the Shapiro-Wilk and Levene tests, respectively.

In the case of the *casadinhos*, an analysis of variance (ANOVA) was performed with type III sum of squares, using the generalized linear model (GLM) procedure. All dependent variables were analysed using a 2-factor ANOVA, specifically the formulation (F) and storage time (ST). In cases where there was a significant interaction between the two factors, the results were compared through the estimated marginal means, in all cases where the effect of each individual factor was statistically significant.

## 4. Conclusions

This work focused on the extraction of phenolic compounds from bio-residues (epicarps) from *Punica granatum* L. to test its potential as a natural functionalising ingredient to be incorporated in a pastry product. The characterization of the phenolic profile of the extracts obtained from the three solvents expressed the existence of fourteen phenolic compounds in ME, the most abundant being the two isomers of punicalagin and galloyl-HHPD-glucose, and seventeen compounds in *Purple Queen*, of which the most abundant were the two isomers of punicalagin, in smaller amounts than those obtained in *Mollar de Elche*. In turn, the presence of anthocyanins, namely cyanidin-3,5-*O*-diglucose, cyanidin-3-*O*-glucose and pelargonidin-3-*O*-glucose, was verified only in *Purple Queen* (being maximum in water extracts). The phenolic composition and the bioactive characteristics presented by the extracts favour the development of applications in the food industry. Thus, given the moderate bioactivity (characteristic required for food application) of the extract obtained from *Purple Queen*, it was incorporated in the formulation of a typical Brazilian pastry product, *casadinhos*. This incorporation has a double application as functional food and as a colouring agent. The incorporation of the natural ingredient did not cause significant changes in the nutritional profile or chemical composition but added antioxidant capacity. The rose colour and soft texture added to the biscuit presented by the new formulations will be pleasant points to present to consumers. This work demonstrates that with the use of eco-friendly solvents and with raw material that until now is considered waste, it is plausible the development of economically interesting products of public interest. Sensory studies that assess the acceptance of this type of food by consumers should also be carried out.

## Figures and Tables

**Table 1:** Retention time (Rt), wavelengths of maximum absorption (λ_max_), mass spectral data and attempt to identify the phenolic compounds.

Peak	R_t_ (min)	λ_máx_ (nm)	Molecular Ion [M − H]^−^ (*m/z*)	MS^2^ (*m/z*)	Identification Attempt
1	4.4	267	331	169(100), 125(33)	Galloylglucose
2	4.6	258, 368	783	481(44), 301(100)	Pedunculagin (bis-HHDP glucose)
3	6.1	256, 378	1083	781(26), 601(13), 301(100)	Punicalagin isomer I
4	7.4	254, 378	1083	781(33), 601(21), 301(100)	Punicalagin isomer II
5	8.9	278	633	463(31), 301(100)	Galloyl-HHDP-glucose
6	13.5	278	785	633(17), 615(5), 483(100), 419(8), 301(50)	Digalloyl-HHDP-glucose
7	15.3	253, 358	463	301(100)	Ellagic acid-hexoside
8	16.6	276	951	933(100), 631(12), 613(9), 463(17), 301(48)	Granatin B (Digalloyl-HHDP-DHHDP-hexose) isomer I
9	17.3	276	951	933(100), 631(15), 613(5), 463(12), 301(49)	Granatin B (Digalloyl-HHDP-DHHDP-hexose) isomer II
10	20.6	256, 364	433	301(100)	Ellagic acid-pentoside
11	21.0	256, 364	447	301(100)	Ellagic acid-rhamnoside
12	22.0	256, 364	301	284(10), 245(5), 185(11), 173(4), 157(6), 145(5)	Ellagic acid
13	23.4	346	593	285(100)	Kaempferol-3-*O*-rutinoside
14	25.0	348	447	285(100)	Kaempferol-3-*O*-glucoside
Anthocyanins					
15	8.9	515	611	449(23), 287(100)	Cyanidin-3,5-*O*-diglucoside
16	16.9	514	449	287(100)	Cyanidin-3-*O*-glucoside
17	20.0	505	433	271(100)	Pelargonidin-3-*O*-glucoside

**Table 2:** Quantification of phenolic compounds (mg/100 g dry weight) in different extracts of pomegranate peel of the *Mollar de Elche* and *Purple Queen* varieties.

Phenolic Compound	Variety of Pomegranate	Type of Extract			Homoscedasticity ^1^ (*p*-Value) (*n* = 27)	ANOVA^2^ (*p*-Value) (*n* = 27)
		Aqueous	Ethanolic	Hydroalcoholic		
Galloylglucose ^A^	*Mollar de Elche*	5.9 ± 0.1 b	8.4 ± 0.4 a	5.7 ± 0.3 b	0.003	<0.001
	*Purple Queen*	1.0 ± 0.1 c	2.1 ± 0.2 a	1.3 ± 0.1 b	<0.001	<0.001
Pedunculagin (bis-HHDP glucose) ^B^	*Mollar de Elche*	3.5 ± 0.4 c	11.8 ± 0.4 a	7.0 ± 0.4 b	0.896	<0.001
	*Purple Queen*	1.2 ± 0.1 c	2.4 ± 0.2 a	1.7 ± 0.1 b	0.217	<0.001
Punicalagin isomer I ^B^	*Mollar de Elche*	23 ± 2 c	61 ± 2 a	40 ± 2 b	0.975	<0.001
	*Purple Queen*	12 ± 1 c	30 ± 1 a	16 ± 1 b	0.348	<0.001
Punicalagin isomer II ^B^	*Mollar de Elche*	18 ± 2 c	73 ± 3 a	28 ± 2 b	0.403	<0.001
	*Purple Queen*	15 ± 1 c	41 ± 3 a	20 ± 1 b	<0.001	<0.001
Galloyl-HHDP-glucose ^B^	*Mollar de Elche*	12 ± 1 c	21 ± 1 a	15 ± 1 b	0.477	<0.001
	*Purple Queen*	2.4 ± 0.2 c	6.6 ± 0.5 a	3.2 ± 0.2 b	<0.001	<0.001
Digalloyl-HHDP-glucose ^B^	*Mollar de Elche*	8 ± 1 c	11 ± 1 a	10 ± 1 b	0.029	<0.001
	*Purple Queen*	1.1 ± 0.1 b	1.7 ± 0.2 a	0.9 ± 0.1 c	0.004	<0.001
Ellagic acid-hexoside ^B^	*Mollar de Elche*	5.2 ± 0.3 c	7.7 ± 0.3 a	4.7 ± 0.3 b	0.936	<0.001
	*Purple Queen*	1.2 ± 0.1 c	3.2 ± 0.2 a	1.7 ± 0.1 b	0.188	<0.001
Granatin B (digalloyl-HHDP- DHHDP-hexose) isomer I^B^	*Mollar de Elche*	8.0 ± 0.5 c	10.6 ± 0.4 a	8.7 ± 0.4 b	0.190	<0.001
	*Purple Queen*	2.8 ± 0.1 c	5.4 ± 0.2 a	3.3 ± 0.2 b	0.403	<0.001
Granatin B (digalloyl-HHDP- DHHDP-hexose) isomer II ^B^	*Mollar de Elche*	2.4 ± 0.3 c	4.3 ± 0.3 a	2.1 ± 0.2 b	0.225	<0.001
	*Purple Queen*	1.1 ± 0.1 b	1.6 ± 0.1 a	0.6 ± 0.1 c	0.337	<0.001
Ellagic acid-pentoside ^B^	*Mollar de Elche*	1.9 ± 0.2 a	2.0 ± 0.2 a	1.5 ± 0.2 b	0.290	<0.001
	*Purple Queen*	0.6 ± 0.1 a	0.7 ± 0.1 a	0.8 ± 0.1 a	<0.001	<0.001
Ellagic acid-rhamnoside ^B^	*Mollar de Elche*	1.3 ± 0.1 c	2.1 ± 0.3 a	0.9 ± 0.2 b	0.043	<0.001
	*Purple Queen*	0.7 ± 0.1	0.8 ± 0.1	0.8 ± 0.1	0.190	0.207
Ellagic acid ^B^	*Mollar de Elche*	4.3 ± 0.2 c	6.2 ± 0.5 a	3.1 ± 0.5 b	0.143	<0.001
	*Purple Queen*	0.7 ± 0.1	0.8 ± 0.1	0.8 ± 0.1	0.009	0.944
Kaempferol-3-*O*-rutinoside ^C^	*Mollar de Elche*	1.3 ± 0.1 a	1.0 ± 0.1 b	0.8 ± 0.1 c	0.652	<0.001
	*Purple Queen*	0.4 ± 0.1 ab	0.3 ± 0.1 c	0.5 ± 0.1 a	0.835	0.013
Kaempferol-3-*O*-glucoside ^D^	*Mollar de Elche* *Purple Queen*	0.5 ± 0.1 a	0.5 ± 0.1 ab	0.4 ± 0.1 b	0.029	0.013
		0.18 ± 0.02 b	0.19 ± 0.04 b	0.27 ± 0.04 a	0.058	0.013
	Anthocyanins					
Cyanidin-3,5-*O*-diglucoside ^E^	*Purple Queen*	7.9 ± 0.4 a	4.5 ± 0.2 c	5.7 ± 0.3 b	0.039	<0.001
Cyanidin-3-*O*-glucoside ^E^	*Purple Queen*	7.3 ± 0.2 a	4.0 ± 0.1 c	5.1 ± 0.3 b	0.097	<0.001
Pelargonidin-3-*O*-glucoside ^F^	*Purple Queen*	4.3 ± 0.2 a	1.8 ± 0.1 c	2.5 ± 0.2 b	0.062	<0.001

Calibration curves: A: ellagic acid (y = 365.2x − 38.923; R^2^ = 0.999); B: gallic acid (γ = 38.466x + 35.44; R^2^ = 0.999); C: kaempferol -3-*O*-rutinoside (y = 182.94x + 96.644; R^2^ = 0.997); D: kaempferol-3-*O*-glucoside (y = 236.33x + 70.006; R^2^ = 0.999); E: cyanidin-3-*O*-glucoside (y = 134578x − 3E6; R^2^ = 0.999); F: pelargonidin-3-*O*-glucoside (y = 61493x − 628875; R^2^ = 0.996). ^1^ *p*-Values less than 0.05 indicate heteroscedastic distributions, and the multiple comparison was made by the Tamhane’s T2 test; *p*-values greater than 0.05 indicate homoscedastic distributions, and the multiple comparison was made by Tukey HSD test. ^2^ If *p*-value is less than 0.05, the corresponding parameter shows significant differences in at least one of the extract types (identified with different letters within the same raw).

**Table 3:** Results of inhibition of the formation of thiobarbituric acid reactive substances (TBARS) and cytotoxicity in different extracts of the epicarp of the *Mollar de Elche* and *Purple Queen* varieties.

Antioxidant and Cytotoxicity	Extract Type			Homoscedasticit ^1^ (*p*-Value) (*n* = 27)	ANOVA ^2^ (*p*-Value) (*n* = 27)
	Aqueous	Ethanol	Hydroalcoholic		
*Mollar de Elche*					
Inhibition of TBARS formation (EC_50_, μg/mL)	10 ± 1 c	18 ± 1 a	16 ± 1 b	0.002	<0.001
PLP2 (GI_50_, μg/mL)	333 ± 9 a	276 ± 12 b	295 ± 34 b	<0.001	<0.001
HeLa (GI_50_, μg/mL)	141 ± 2 b	92 ± 3 c	178 ± 5 a	0.042	<0.001
HepG2 (GI_50_, μg/mL)	196 ± 6 b	111 ± 4 c	228 ± 4 a	0.153	<0.001
MCF7 (GI_50_, μg/mL)	178 ± 4 b	122 ± 6 c	216 ± 6 a	0.191	<0.001
NCI-H460 (GI_50_, μg/mL)	211 ± 16 b	194 ± 3 c	259 ± 5 a	<0.001	<0.001
*Purple Queen*					
Inhibition of TBARS formation (EC_50_, μg/mL)	29 ± 1 c	37 ± 1 a	19 ± 3 b	0.001	<0.001
PLP2 (GI_50_, μg/mL)	354 ± 24 a	299 ± 12 b	357 ± 7 b	0.012	<0.001
HeLa (GI_50_, μg/mL)	223 ± 6 b	153 ± 6 c	321 ± 13 a	0.010	<0.001
HepG2 (GI_50_, μg/mL)	205 ± 5 b	216 ± 2 c	318 ± 6 a	0.026	<0.001
MCF7 (GI_50_, μg/mL)	246 ± 13 b	228 ± 8 c	269 ± 6 a	0.030	<0.001
NCI-H460 (GI_50_, μg/mL)	260 ± 11 b	268 ± 36 c	250 ± 14 a	0.001	0.293

^1^ Values of *p* less than 0.05 indicate heteroscedastic distributions, the multiple comparison being made by the Tamhane’s T2 test; *p*-values greater than 0.05 indicate homoscedastic distributions, and multiple comparisons were made by the Tukey HSD test. ^2^ If the *p*-value is lower than 0.05, the corresponding parameter presents significant differences in at least one of the types of extract (identified with different letters within the same raw).

**Table 4:** Results of the antibacterial activity of different extracts of the pomegranate epicarp of the varieties *Mollar de Elche* and *Purple Queen*.

Bacteria Strain	Variety of Pomegranate	Extract type			Ampicillin (20 mg/mL)	Imipenem (1 mg/mL)
		Aqueous (MIC)^A^	Ethanol (MIC) ^A^	Hydroalcoholic (MIC) ^A^		
Gram-negative bacteria						
*Escherichia coli*	*Mollar de Elche*	2.5	1.25	2.5	MIC < 0.15	MIC < 0.0078
	*Purple Queen*	2.5	1.25	1.25	MBC < 0.15	MBC < 0.0078
*Klebsiella pneumoniae*	*Mollar de Elche*	2.5	2.5	1.25	MIC = 10	MIC < 0.0078
	*Purple Queen*	5	2.5	2.5	MBC = 20	MBC < 0.0078
*Morganella morganii*	*Mollar de Elche*	1.25	1.25	2.5	MIC = 20	MIC < 0.0078
	*Purple Queen*	2.5	1.25	1.25	MBC > 20	MBC < 0.0078
*Proteus mirabilis*	*Mollar de Elche*	1.25	1.25	2.5	MIC < 0.15	MIC < 0.0078
	*Purple Queen*	2.5	1.25	1.25	MBC < 0.15	MBC < 0.0078
*Pseudomonas aeruginosa*	*Mollar de Elche*	10	10	10	MIC > 20	MIC = 0.5
	*Purple Queen*	20	10	10	MBC > 20	MBC = 1
Gram-positive bacteria						
*Enterococcus faecalis*	*Mollar de Elche*	1.25	0.625	0.625	MIC < 0.15	nt
	*Purple Queen*	2.5	2.5	2.5	MBC < 0.15	
*Listeria monocytogenes*	*Mollar de Elche*	5	2.5	2.5	MIC < 0.15	MIC < 0.0078
	*Purple Queen*	5	5	5	MBC < 0.15	MBC < 0.0078
*MRSA*	*Mollar de Elche*	0.625	0.625	0.625	MIC < 0.15	nt
	*Purple Queen*	1.25	0.625	0.625	MBC < 0.15	

A: The minimum bactericidal concentration (MBC) values for the extracts were not presented because this activity was not verified until the maximum concentration tested (20 mg/mL). MIC: minimum inhibitory concentration. MBC: minimum bactericidal concentration. MRSA: Multi-resistant *Staphylococcus aureus*.

**Table 5:** Nutritional composition, free sugars (g/100 g), energy value (kcal/100 g), profile in fatty acids, colorimetric parameters and textural characteristics of the different formulations of *casadinhos*.

Factors Analyzed		Water		Fat		Proteins		Carbohydrates		Fructose		Glucose		Sucrose		Energy	Ashes
Formulation(F)	CT	3 ± 1		21 ± 1		0.7 ± 0.1 c		75 ± 1		4.1 ± 0.2		4 ± 1		39 ± 1		494 ± 5	0.5 ± 0.1
	CEC	7 ± 1		19 ± 1		0.8 ± 0.1 b		73 ± 1		5.8 ± 0.5		7 ± 1		38 ± 1		469 ± 8	0.5 ± 0.1
	CECP	4 ± 1		21 ± 1		0.9 ± 0.1 a		73 ± 1		5.8 ± 0.5		6 ± 1		32 ± 3		491 ± 4	0.5 ± 0.1
*p*-Value (*n* = 27) ^A^		<0.001		<0.001		<0.001		<0.001		<0.001		<0.001		<0.001		<0.001	<0.001
Storage time (ST)	0 days	6 ± 2		21 ± 1		0.9 ± 0.1		72 ± 1		5 ± 1		6 ± 1		35 ± 5		478 ± 14	0.4 ± 0.1
	7 days	4 ± 2		21 ± 1		0.8 ± 0.1		74 ± 1		5 ± 1		6 ± 1		36 ± 4		485 ± 12	0.6 ± 0.1
	14 days	3 ± 1		21 ± 1		0.8 ± 0.1		74 ± 1		5 ± 1		5 ± 1		38 ± 2		490 ± 9	0.5 ± 0.1
*p*-Value (*n* = 27) ^B^		<0.001		0.134		0.166		<0.001		0.262		0.212		0.006		<0.001	<0.001
F × ST *p*-value (*n* = 81) ^A^		<0.001		0.016		0.103		0.013		0.002		<0.001		<0.001		<0.001	<0.001
		**C6:0**	**C8:0**	**C10:0**	**C12:0**	**C14:0**	**C14:1**	**C15:0**	**C16:0**	**C16:1**	**C18:0**	**C18:1n9**	**C18:3n6**	**C18:3n3**	**SFA**	**MUFA**	**PUFA**
Formulation (F)	CT	3 ± 1	2.0 ± 0.4	3.7 ± 0.4	4.4 ± 0.3	12 ± 1	1.0 ± 0.1	1.0 ± 0.1	30 ± 1	1.4 ± 0.1	10.9 ± 0.3	21 ± 1	6.1 ± 0.5	0.9 ± 0.1	69 ± 2	24 ± 2	7.2 ± 0.5
	CEC	4 ± 1	1.9 ± 0.2	3.5 ± 0.2	4.3 ± 0.3	12 ± 1	1.0 ± 0.1	1.0 ± 0.1	31 ± 1	1.4 ± 0.1	10.9 ± 0.3	21 ± 1	6.0 ± 0.5	0.9 ± 0.1	69 ± 1	23 ± 1	7.1 ± 0.5
	CECP	3 ± 1	1.8 ± 0.3	3.4 ± 0.4	4.2 ± 0.3	12 ± 1	0.8 ± 0.1	1.0 ± 0.1	31 ± 1	1.4 ± 0.1	11.2 ± 0.5	21 ± 1	6.6 ± 0.5	1.0 ± 0.1	69 ± 2	24 ± 1	7.8 ± 0.5
*p*-Value (*n* = 27) ^A^		0.166	0.084	0.015	0.057	0.128	<0.001	0.676	0.135	0.120	0.007	0.219	<0.001	0.202	0.155	0.817	<0.001
Storage time (ST)	0 days	3 ± 1	1.8 ± 0.2	3.4 ± 0.3	4.4 ± 0.2	12.2 ± 0.5	0.9 ± 0.1	1.0 ± 0.1	31 ± 1	1.4 ± 0.1	10.9±0.3	21 ± 1	6.3 ± 0.3	1.0 ± 0.1	69 ± 2	24 ± 2	7.3 ± 0.3
	7 days	3 ± 1	1.9 ± 0.4	3.5 ± 0.5	4.4 ± 0.3	12.2 ± 0.4	1.0 ± 0.1	1.0 ± 0.1	31 ± 1	1.5 ± 0.1	11.1±0.4	21 ± 1	5.7 ± 0.5	0.9 ± 0.1	69 ± 2	24 ± 1	6.8 ± 0.5
	14 days	4 ± 1	2.1 ± 0.1	3.6 ± 0.2	4.1 ± 0.2	11.4 ± 0.5	0.9 ± 0.1	1.0 ± 0.1	30 ± 1	1.4 ± 0.1	11.0±0.4	20 ± 1	6.7 ± 0.5	1.0 ± 0.1	69 ± 1	23 ± 1	7.9 ± 0.5
*p*-Value (*n* = 27) ^B^		<0.001	0.001	0.048	<0.001	<0.001	0.006	0.061	<0.001	<0.001	0.361	0.012	<0.001	<0.001	0.155	0.511	<0.001
F × ST *p*-value (*n* = 81) ^A^		<0.001	<0.001	<0.001	<0.001	<0.001	0.002	0.355	0.002	<0.001	<0.001	<0.001	<0.001	<0.001	<0.001	<0.001	<0.001
		L*		a*		b*		**Hardness**		**Fracturability**		**Chewiness**			**Resilience**		
Formulation (F)	CT	78 ± 1		−1 ± 1		30 ± 1		4614 ± 685		4571 ± 828		172 ± 110			0.004 ± 0.002		
	CEC	65 ± 2		8 ± 1		28 ± 1		1456 ± 252		1394 ± 311		29 ± 5			0.019 ± 0.003		
	CECP	64 ± 1		8 ± 1		30 ± 1		2883 ± 187		2932 ± 152		46 ± 13			0.023 ± 0.002		
*p*-Value (n = 27)^A^		<0.001		<0.001		<0.001		<0.001		<0.001		<0.001			<0.001		
Storage time (ST)	0 days	68 ± 7		6 ± 5		30 ± 2		2553 ± 1121		2439 ± 1043		46 ± 16			0.02 ± 0.01		
	7 days	70 ± 6		5 ± 4		29 ± 2		2972 ± 1288		3046 ± 1474		77 ± 51			0.02 ± 0.01		
	14 days	70 ± 7		5 ± 4		29 ± 2		3428 ± 1564		3413 ± 1511		124 ± 138			0.01 ± 0.01		
*p*-Value (*n* = 27) ^B^		0.421		0.580		0.670		0.061		0.034		0.004			0.241		
F × ST *p*-Value (*n* = 81) ^A^		0.010		<0.001		<0.001		<0.001		<0.001		<0.001			<0.001		

^A^*p*-Values lower than 0.05 indicate that at least one of the formulations is significantly different from the others. ^B^*p*-Values less than 0.05 indicate that at least one of the storage times is significantly different from the remaining ones. ^C^*p*-Values less than 0.05 indicate a significant interaction between the factors, so it is not possible to classify the differences induced by each of the individual factors.

## References

[1] Gullón B., Garrote G., Alonso J.L., Parajó J.C. (2007). Production of L-lactic acid and oligomeric compounds from apple pomace by simultaneous saccharification and fermentation: A response surface methodology assessment. J. Agric. Food Chem..

[2] Barbulova A., Colucci G., Apone F. (2015). New Trends in Cosmetics: By-Products of Plant Origin and Their Potential Use as Cosmetic Active Ingredients. Cosmetic.

[3] Huang Y.-L., Shi S.-H. (2002). Phylogenetics of Lythraceae sensu lato: A Preliminary Analysis Based on Chloroplast rbc L Gene, psa A - ycf 3 Spacer, and Nuclear rDNA Internal Transcribed Spacer (ITS) Sequences. Int. J. Plant Sci..

[4] Fawole O.A., Makunga N.P., Opara U.L. (2012). Antibacterial, antioxidant and tyrosinase-inhibition activities of pomegranate fruit peel methanolic extract. BMC Complement. Altern. Med..

[5] Fischer U.A., Carle R., Kammerer D.R. (2011). Identification and quantification of phenolic compounds from pomegranate (*Punica granatum* L.) peel, mesocarp, aril and differently produced juices by HPLC-DAD-ESI/MS. Food Chem..

[6] Mena P., Calani L., Dall’Asta C., Galaverna G., García-Viguera C., Bruni R., Del Rio D. (2012). Rapid and comprehensive evaluation of (Poly)phenolic compounds in pomegranate (*Punica granatum* L.) Juice by UHPLC-MSn. Molecules.

[7] Çam M., Hişil Y. (2010). Pressurised water extraction of polyphenols from pomegranate peels. Food Chem..

[8] Yaich H., Cheikhrouhou S., Ayadi M.A., Khemakhem I., Attia H., Abid M., Bouaziz M. (2017). Antioxidant properties and phenolic profile characterization by LC–MS/MS of selected Tunisian pomegranate peels. J. Food Sci. Technol..

[9] Lansky E.P., Newman R.A. (2007). *Punica granatum* (pomegranate) and its potential for prevention and treatment of inflammation and cancer. J. Ethnopharmacol..

[10] Esmaillzadeh A., Tahbaz F., Gaieni I., Alavi-Majd H., Azadbakht L. (2006). Cholesterol-lowering effect ot concentrated pomegranate juice consumption in type II diabetic patients with hyperlipidemia. Int. J. Vitam. Nutr. Res..

[11] Bagri P., Ali M., Aeri V., Bhowmik M., Sultana S. (2009). Antidiabetic effect of *Punica granatum* flowers: Effect on hyperlipidemia, pancreatic cells lipid peroxidation and antioxidant enzymesin experimental diabetes. Food Chem. Toxicol..

[12] Braidy N., Selvaraju S., Essa M.M., Vaishnav R., Al-Adawi S., Al-Asmi A., Alobaidy A., Lakhtakia R., Guillemin G.J. (2013). Neuroprotective effects of a variety of pomegranate juice extracts against MPTP-induced cytotoxicity and oxidative stress in human primary neurons. Oxid. Med. Cell. Longev..

[13] Yang X., Nisar T., Hou Y., Gou X., Sun L., Guo Y. (2018). Pomegranate peel pectin can be used as an effective emulsifier. Food Hydrocolloids.

[14] Gómez-Caravaca A.M., Verardo V., Toselli M., Segura-Carretero A., Alberto Fernandez-Gutierrez A., Caboni M.F. (2013). Determination of the Major Phenolic Compounds in Pomegranate Juices by HPLC−DAD−ESI-MS. J. Agric. Food Chem..

[15] Ambigaipalan P., de Camargo A.C., Shahidi F. (2017). Identification of phenolic antioxidants and bioactives of pomegranate seeds following juice extraction using HPLC-DAD-ESI-MSn. Food Chem..

[16] Ambigaipalan P., De Camargo A.C., Shahidi F. (2016). Phenolic Compounds of Pomegranate Byproducts (Outer Skin, Mesocarp, Divider Membrane) and Their Antioxidant Activities. J. Agric. Food Chem..

[17] Brighenti V., Groothuis S.F., Prencipe F.P., Amir R., Benvenuti S., Pellati F. (2017). Metabolite fingerprinting of *Punica granatum* L. (pomegranate) polyphenols by means of high-performance liquid chromatography with diode array and electrospray ionization-mass spectrometry detection. J. Chromatogr. A.

[18] He L., Xu H., Liu X., He W., Yuan F., Hou Z., Gao Y. (2011). Identification of phenolic compounds from pomegranate (*Punica granatum* L.) seed residues and investigation into their antioxidant capacities by HPLC–ABTS+ assay. Food. Res. Int..

[19] Nuncio-Jáuregui N., Nowicka P., Hernández S.M.-P.F., Wojdyło Á.A.C.-B.A. (2015). Identification and quantification of major derivatives of ellagic acid and antioxidant properties of thinning and ripe Spanish pomegranates. J. Funct..

[20] Qu W., Breksa A.P., Pan Z., Ma H. (2012). Quantitative determination of major polyphenol constituents in pomegranate products. Food Chem..

[21] Wafa B.A., Makni M., Ammar S., Khannous L., Hassana A., Bouaziz M., Gdoura R. (2017). Antimicrobial effect of the Tunisian Nana variety *Punica granatum* L. extracts against *Salmonella enterica* (serovars Kentucky and Enteritidis) isolated from chicken meat and phenolic composition of its peel extract. Int. J. Food Microbiol..

[22] Alexandre E.M.C., Silva S., Santos S.A.O., Silvestre A.J.D., Duarte M.F., Saraiva J.A., Pintado M. (2019). Antimicrobial activity of pomegranate peel extracts performed by high pressure and enzymatic assisted extraction. Food Res. Int..

[23] Derakhshan Z., Ferrante M., Tadie M., Ansari F., Heydarif A., Hosseinig M.S., Contid G.O., Sadrabad E.K. (2018). Antioxidant activity and total phenolic content of ethanolic extract of pomegranate peels, juice and seeds. Food Chem. Toxicol..

[24] Gullon B., Pintado M.E., Perez-Alvarez J.A., Viuda-Martos M. (2016). Assessment of polyphenolic profile and antibacterial activity of pomegranate peel (*Punica granatum*) flour obtained from co-product of juice extraction. Food Control.

[25] Caleja C., Barros L., Antonio A.L., Oliveira M.B.P.P., Ferreira I.C.F.R. (2017). A comparative study between natural and synthetic antioxidants: Evaluation of their performance after incorporation into biscuits. Food Chem..

[26] Caleja C., Barros L., Barreira J.C.M., Ciric A., Sokovic M., Calhelha R.C., Oliveira M.B.P.P., Ferreira I.C.F.R. (2018). Suitability of *Melissa officinalis* L. extract rich in rosmarinic acid as a potential enhancer of functional properties in newly developed cupcakes. Food Chem..

[27] Wang W.-Q., Bao Y.-H., Chen Y. (2013). Characteristics and antioxidant activity of water-soluble Mail lard reaction products from interactions in a whey protein isolate and sugars system. Food Chem..

[28] Takwa S., Caleja C., Barreira J.C.M., Soković M., Achour L., Barros L., Ferreira I.C.F.R. (2018). *Arbutus unedo* L. and *Ocimum basilicum* L. as sources of natural preservatives for food industry: A case study using loaf bread. LWT.

[29] López C.J., Caleja C., Prieto M.A., Barreiro M.F., Barros L., Ferreira I.C.F.R. (2018). Optimization and comparison of heat and ultrasound assisted extraction techniques to obtain anthocyanins from *Arbutus unedo* L. fruits. Food Chem..

[30] Bessada S.M., Barreira J.C.M., Barros L., Ferreira I.C.F.R., Oliveira M.B.P.P. (2016). Phenolic profile and antioxidant activity of *Coleostephus myconis* (L.) Rchb. f.: An underexploited and highly disseminated species. Ind. Crop Prod..

[31] Gonçalves G.A., Soares A.A., Correa R.C.G., Barros L., Haminiuk C.W.I., Peralta R.M., Bracht A. (2017). Merlot grape pomace hydroalcoholic extract improves the oxidative and inflammatory states of rats with adjuvant-induced arthritis. J. Funct. Foods.

[32] Barros L., Pereira E., Calhelha R.C., Dueñas M., Carvalho A.M., Santos-Buelga C., Ferreira I.C.F.R. (2013). Bioactivity and chemical characterization in hydrophilic and lipophilic compounds of *Chenopodium ambrosioides* L.. J. Funct. Foods.

[33] Guimarães R., Barros L., Dueñas M., Carvalho A.M., Queiroz M.J.R.P., Santos-Buelga C., Ferreira I.C.F.R. (2013). Characterisation of phenolic compounds in wild fruits from Northeastern Portugal. Food Chem..

[34] Pires T.C.S.P., Dias M.I., Barros L., Alves M.J., Oliveira M.B.P.P., Santos-Buelga C., Ferreira I.C.F.R. (2018). Antioxidant and antimicrobial properties of dried Portuguese apple variety (*Malus domestica* Borkh. Cv Bravo de Esmolfe). Food Chem..

[35] Caleja C., Barros L., Antonio A.L., Carocho M., Oliveira M.B.P.P., Ferreira I.C.F.R. (2016). Fortification of yogurts with different antioxidant preservatives: A comparative study between natural and synthetic additives. Food Chem..

[36] Latimer G.W., AOAC (2016). AOAC Official Methods of Analysis.

